# Longitudinal monitoring of tau aggregation in progressive supranuclear palsy with [^18^F]PI‐2620 PET

**DOI:** 10.1002/alz.71195

**Published:** 2026-02-24

**Authors:** Johannes Gnörich, Julia Kusche‐Palenga, Carla Palleis, Antonia Neubauer, Lukas Frontzkowski, Alexander Jäck, Agnes Kling, Theresa Bauer, Hannah Eyob, Katharina Probst, Sebastian N. Roemer‐Cassiano, Alexander M. Bernhardt, Sabrina Katzdobler, Lena Marth, Mirlind Zaganjori, Franziska Hopfner, Andreas Zwergal, Jan Häckert, Michael Rullmann, Osama Sabri, Henryk Barthel, Sophia Stöcklein, Rudolf A. Werner, Mikael Simons, Johannes Levin, Jochen Herms, Nicolai Franzmeier, Günter U. Höglinger, Matthias Brendel, for the German Imaging Initiative for Tauopathies (GII4T)

**Affiliations:** ^1^ Department of Nuclear Medicine LMU University Hospital LMU Munich Munich Germany; ^2^ German Center for Neurodegenerative Diseases (DZNE) Munich Munich Germany; ^3^ Department of Neurology LMU University Hospital LMU Munich Munich Germany; ^4^ Munich Cluster for Systems Neurology (SyNergy) Munich Germany; ^5^ Center for Neuropathology and Prion Research LMU University Hospital LMU Munich Munich Germany; ^6^ Institute for Stroke and Dementia Research LMU Hospital LMU Munich Munich Germany; ^7^ German Center for Vertigo and Balance Disorders, DSGZ LMU University Hospital LMU Munich Munich Germany; ^8^ Department of Psychiatry Psychotherapy and Psychosomatics Medical Faculty University of Augsburg BKH Augsburg Augsburg Germany; ^9^ Department of Nuclear Medicine University Hospital Leipzig Leipzig Germany; ^10^ Department of Nuclear Medicine Municipal Hospital Dessau Dessau‐Roßlau Germany; ^11^ Department of Radiology LMU Hospital LMU Munich Munich Germany; ^12^ The Russell H. Morgan Department of Radiology and Radiological Sciences Division of Nuclear Medicine Johns Hopkins School of Medicine Baltimore Maryland USA; ^13^ Department of Psychiatry and Neurochemistry University of Gothenburg, The Sahlgrenska Academy, Institute of Neuroscience and Physiology Mölndal and Gothenburg Sweden

**Keywords:** 4‐repeat tauopathies, disease monitoring, progressive supranuclear palsy, tau positron emission tomography, tau spreading

## Abstract

**INTRODUCTION:**

Progressive supranuclear palsy (PSP), a 4‐repeat tauopathy, can be visualized using [^18^F]PI‐2620 tau positron emission tomography (PET). However, the value of sequential [^18^F]PI‐2620 imaging for tracking tau accumulation during the disease course has not yet been investigated.

**METHODS:**

Twenty‐three PSP patients underwent two [^18^F]PI‐2620 PET scans (interval: 21.4 ± 4.3 months) and were compared to cross‐sectional data from 25 healthy controls. Regional volume of distribution ratio values were analyzed for longitudinal tau changes, clinical correlations, and network‐based propagation. *Post mortem* analyses examined neuronal density and AT8 tau pathology.

**RESULTS:**

Subcortical tau PET signals increased, strongest in the globus pallidus internus (*P* < 0.0001). Patients with low baseline tau showed the largest increases. Despite clinical worsening (Progressive Supranuclear Palsy Rating Scale +48%), tau PET change did not correlate with symptom progression. Tau accumulation followed functional connectivity (*R* = 0.34, *P* < 0.0001). *Post mortem* data linked elevated tau PET to higher AT8 burden despite neuronal loss.

**DISCUSSION:**

[^18^F]PI‐2620 PET enables monitoring of tau progression in PSP, indicating network‐based tau propagation with saturation in advanced stages.

## Highlights

- Longitudinal tau positron emission tomography (PET) reveals subcortical tau progression in progressive supranuclear palsy over 21 months.
- Tau accumulation shows a non‐linear trajectory with ceiling effects .
- PET signal remains elevated despite neuronal loss due to residual tau pathology.
- Functional connectivity predicts similarity in tau accumulation rates across regions.

## 1 BACKGROUND

Tau protein aggregation is a central feature of multiple neurodegenerative disorders, yet the molecular forms and distribution of tau pathology differ substantially between disease entities. Alzheimer's disease (AD), the most common cause of dementia, is characterized by neurofibrillary tangles (NFTs) composed of both 3‐repeat (3R) and 4‐repeat (4R) tau isoforms, accompanied by extracellular amyloid beta (Aβ) deposits.^1^ In AD, the spatiotemporal progression of tau pathology has been well studied through autopsy‐based Braak staging schemes^2^ and, more recently, by positron emission tomography (PET) using tau radiotracers.^3^, ^4^, ^5^ Such tau PET studies have revealed that the extent of cortical tau deposition correlates strongly with neurodegeneration and cognition, positioning tau accumulation as a promising biomarker to assess disease progression and treatment efficacy.^6^, ^7^, ^8^, ^9^

In contrast, progressive supranuclear palsy (PSP) and corticobasal degeneration (CBD) collectively termed primary 4‐repeat tauopathies (4RT) show predominantly 4R tau aggregates in astrocytic and neuronal structures^10^, ^11^ and exhibit clinical manifestations that differ substantially from those observed in AD. In particular, PSP presents with postural instability, vertical gaze palsy, and a rapid disease course culminating in significant disability,^12^, ^13^ while CBD, which often presents clinically as corticobasal syndrome (CBS), is characterized by asymmetric motor symptoms, apraxia, cortical sensory deficits, and progressive cognitive decline.^14^, ^15^ However, diagnostic accuracy is often hampered by the overlap of early clinical symptoms with those of other neurodegenerative conditions. Current clinical diagnostic criteria incorporate structural and metabolic imaging, yet biomarkers with sufficient sensitivity and specificity for 4RT, especially at early stages, remain elusive.^13^

Recent advances in tau PET imaging, including second‐generation radiotracers such as [^18^F]PI‐2620, hold promise for improved detection of 4R tau pathology in living patients with PSP.^16^, ^17^ In vitro and in vivo evidence suggests that [^18^F]PI‐2620 exhibits affinity for 4R tau aggregates, with less off‐target binding than older‐generation tau ligands.^18^, ^19^ Although first cross‐sectional studies have shown that [^18^F]PI‐2620 can distinguish PSP from healthy controls and disease controls, the utility of this tracer for longitudinal monitoring of disease progression in PSP has yet to be fully established. This question is clinically relevant, as established imaging readouts, including atrophy and reduced glucose metabolism, often represent relatively late manifestations in PSP, whereas tau PET may have the potential to detect earlier pathologic changes and track disease evolution. Therefore, establishing robust, longitudinal 4R tau PET biomarkers could aid in earlier diagnosis, disease monitoring, and evaluation of targeted treatments aimed at reducing tau burden.

Here, we report on the first longitudinal [^18^F]PI‐2620 PET study in individuals with PSP. Drawing on the clinical gaps and biomarker criteria previously outlined for PSP, we aimed to examine whether repeated [^18^F]PI‐2620 imaging over time can capture progressive changes in 4R tau burden and thereby serve as an effective marker of disease evolution. To elucidate how tau pathology spreads across connected brain regions, we assessed whether longitudinal [^18^F]PI‐2620 changes follow intrinsic functional connectivity derived from resting‐state functional magnetic resonance imaging (fMRI), testing a network‐based propagation model of 4R tau pathology. In addition, *post mortem* histopathological analyses were performed in PSP brains to validate in vivo tracer binding and link regional PET signals to underlying tau burden. Together, this multimodal design integrates molecular imaging, network analysis, and neuropathology to provide a comprehensive framework for assessing disease mechanisms and progression in 4RT.

## 2 METHODS

### 2.1 Investigated population and clinical assessments

All subjects were scanned at the Ludwig‐Maximilians‐University (LMU) University Hospital, Munich Department of Nuclear Medicine between February 2018 and February 2025. Patients with PSP were diagnosed by movement disorders specialists at the Department of Neurology, LMU University Hospital, according to current Movement Disorder Society (MDS)‐PSP diagnostic criteria,^13^ and only those with negative cerebrospinal fluid (CSF; Aβ_42/40_ ratio < 5.5) and/or PET Aβ biomarkers were included along with age‐matched, Aβ‐negative, cognitive unimpaired healthy controls (Table 1).^20^ The longitudinal cohort was not selectively enriched for specific PSP subtypes. Instead, it comprised all individuals from original cross‐sectional datasets who fulfilled MDS‐PSP spectrum criteria at both baseline and follow‐up and who were able and willing to return for repeat PET imaging after a minimum planned interval of 12 months (actual minimum interval: 14 months). Aβ PET status was determined visually on late‐phase (90–110 minute p.i.) scans acquired with [^18^F]flutemetamol or [^18^F]florbetaben by three experienced nuclear physicians following manufacturer guidelines (Life Molecular Imaging for NeuraCeq, GE HealthCare for Vizamyl) as described previously.^20^ In participants for whom both CSF and Aβ PET data were available, all results were concordantly negative, and no PET/CSF discordance was observed in either the PSP or healthy control groups. Disease duration was defined as the interval between symptom onset, as reported by the patient or a close caregiver, and the time of first PET imaging. The Progressive Supranuclear Palsy Rating Scale (PSPRS) ^21^ and the motor part of the Unified Parkinson's Disease Rating Scale (UPDRS‐III) ^22^ were used to gauge disease severity, whereas the Montreal Cognitive Assessment (MoCA) or converted Mini‐Mental State Examination (MMSE) score was used to assess cognitive impairment.^23^ MoCA scores were converted to MMSE values using this validated equipercentile conversion table, allowing harmonized comparison of cognitive performance across visits; in total, 11 of 46 cognitive assessments in the PSP cohort required such conversion. Additionally, Schwab and England Activities of Daily Living scores (SEADL) were recorded as a global measure of functional ability. All participants (or their legal representatives) provided written consent for PET imaging. The study protocol and PET data analyses were approved by the local ethics committee (LMU Munich, application numbers 17‐569 and 19‐022). The tissue samples from all the autopsy cases of our previously published cohort, consisting of seven patients with pathologically confirmed PSP, were provided by Neurobiobank Munich, LMU Munich.^19^ They were collected according to the guidelines of the local ethics committee, and the usage of the material for this project was additionally approved (application number 19‐244). The study was carried out according to the principles of the Declaration of Helsinki and its later amendments or comparable ethical standards.

**Research in context**

1. **Systematic Review**: We conducted a literature review using PubMed and recent conference proceedings to identify prior tau positron emission tomography (PET) studies in primary tauopathies. While cross‐sectional studies exist, longitudinal investigations using second‐generation tracers targeting 4R tau are still lacking.
2. **Interpretation**: Our study demonstrates progressive subcortical tau accumulation in progressive supranuclear palsy (PSP) using serial [^18^F]PI‐2620 tau PET, following a non‐linear trajectory with ceiling effects. Tau accumulation was associated with functional brain connectivity, and *post mortem* analyses revealed that elevated tau burden persists despite neuronal loss, suggesting that tau PET can detect substantial pathology even in advanced disease stages.
3. **Future Directions**: Future studies should validate tau PET as a progression marker across different PSP subtypes and investigate its predictive utility in larger cohorts. Moreover, longitudinal [^18^F]PI‐2620 tau PET should be further evaluated as a biomarker in therapeutic trials targeting 4R tauopathies.

**TABLE 1:** Demographics at the group level.

Demographics	PSP	Healthy controls
No.	23	25
Subgroups	PSP‐RS (*n* = 9), PSP‐CBS (*n* = 7), PSP‐F (*n* = 2), PSP‐*P* (*n* = 3), PSP‐PGF (*n* = 2)	NA
Age at baseline, mean (SD), y	69.0 (± 6.5)	70.3 (± 8.5)
Disease duration, mean (SD), months	23.7 (± 15.5)	NA
**Sex, no. (%)**		
Female	6 (26)	12 (48)
Male	17 (74)	13 (52)
Follow‐up [^18^F]PI‐2620 PET interval, months	21.4 (± 4.3)	27.0 (*n* = 1)
Aβ PET, no.	12	18
CSF Aβ_42/40,_ no.	13	15
**Clinical measures (BL/FU)**		
MoCA score, mean (SD)	23.6 (± 4.1) / 21.0 (± 6.8)	29.5 (± 1.0)
MMSE score, mean (SD)	26.5 (± 3.6) / 25.4 (± 5.5)	29.0 (± 1.5)
PSPRS score, mean (SD)	23.7 (± 9.5) / 33.8 (± 13.6)	NA
UPDRS‐III score, mean (SD)	35.4 (± 13.1) / 47.6 (± 13.6)	NA
SEADL score, mean (SD)	69.1 (± 13.1) / 54.1 (± 16.8)	NA

Abbreviations: Aβ, amyloid beta; BL, baseline; CSF, cerebrospinal fluid; FU, follow‐up; MMSE, Mini‐Mental State Examination; MoCA, Montreal Cognitive Assessment; NA,  not applicable; PET, positron emission tomography; PSP,  progressive supranuclear palsy; PSP‐CBS, progressive supranuclear palsy with corticobasal syndrome; PSP‐F, progressive supranuclear palsy with frontal presentation; PSP‐P, progressive supranuclear palsy with predominant Parkinsonism; PSP‐PGF, progressive supranuclear palsy with progressive gait freezing; PSPRS, progressive supranuclear palsy rating system; PSP‐RS, progressive supranuclear palsy with Richardson's syndrome; SD, standard deviation; SEADL, Schwab and England Activities of Daily Living Scale; UPDRS‐III, Unified Parkinson Disease Rating Scale.

### 2.2 Radiosynthesis

Radiosynthesis of [^18^F]PI‐2620 was achieved by nucleophilic substitution on a BOC‐protected nitro precursor using an automated synthesis module (IBA, Synthera). The protecting group was cleaved under the radiolabeling conditions. The product was purified by semipreparative high‐performance liquid chromatography. Radiochemical purity was 99%. Non‐decay corrected yields were ≈ 35% with a molar activity of 8∙10^6^ GBq/mmol at the end of synthesis.

### 2.3 Tau PET imaging

All patients were scanned at the Department of Nuclear Medicine, LMU University Hospital, with a Biograph 64 or a Siemens mCT PET/computed tomography (CT) scanner (both Siemens). For most longitudinal participants, baseline and follow‐up scans were obtained on the same scanner to minimize potential bias from inter‐scanner variability. Both scanners were accredited by the EANM Research Ltd. (EARL) program, ensuring standardized quantitative performance and harmonized reconstruction parameters across systems (https://earl.eanm.org/). In brief, the dynamic brain PET data were acquired in 3‐dimensional list‐mode over 60 minutes and reconstructed into a 336 × 336 × 109 matrix (voxel size: 1.02 × 1.02 × 2.03 mm^3^) using the built‐in ordered subset expectation maximization (OSEM) algorithm with 4 iterations, 21 subsets, and a 5 mm Gaussian filter on the Siemens Biograph and with 5 iterations, 24 subsets, and a 5 mm Gaussian filter on the Siemens mCT. A low‐dose CT scan preceded the PET acquisition and served as attenuation correction. Frame binning (*n* = 35) was standardized to 12 × 5 seconds, 6 × 10 seconds, 3 × 20 seconds, 7 × 60 seconds, 4 × 300 seconds, and 3 × 600 seconds.

### 2.4 Tau PET data analysis

[^18^F]PI‐2620 volume of distribution ratio (VTr) values were calculated with image‐derived input functions (IDIF) using Logan plots as previously described.^24^, ^25^ Our recent validation study has shown that non‐invasive IDIF‐based Logan modeling provides reliable estimates of [^18^F]PI‐2620 binding, with performance comparable to arterial input function‐based approaches in PSP.^24^ In brief, after initial motion correction of each full dynamic dataset, IDIF were obtained through manual extraction of the PET signal from the carotid artery during the 60 minute dynamic PET scan. For manual extraction, the blood activity concentration in the bilateral carotid artery was detected in early frames of the dynamic PET images, and spheres with a diameter of 5.0 mm were placed as volumes of interest (VOI) in the pars cervicalis of the internal carotid artery prior to entering the pars petrosal using PMOD version 4.2 (PMOD Technologies). The activity concentration over time was calculated with the average and the five highest voxel intensity values of the VOI. All images were registered to Montreal Neurology Institute (MNI) space using the established [^18^F]PI‐2620 PET template.^26^

The inferior cerebellum as well as a composite of temporal and orbital white matter served as reference tissues for modeling, selected based on prior comprehensive validation in dynamic [^18^F]PI‐2620 PET, demonstrating that deeper white matter contains minimal 4R tau pathology and exhibits markedly reduced [^18^F]PI‐2620 binding compared to gray matter and the gray matter and white matter boundary.^19^, ^25^, ^27^ The temporo‐orbital white matter provided the most stable and pathology‐poor normalization region, outperforming conventional inferior cerebellar references in PSP with regard to diagnostic group separation and clinical–PET associations.^25^ To ensure comparable processing and to address potential bias, analyses using inferior cerebellum were additionally performed. This normalization step is critical for longitudinal tracking of tau accumulation, as it helps to minimize inter‐scan variability and ensures that increases in signal reflect true pathological progression rather than non‐specific changes.

Finally, [^18^F]PI‐2620 VTr values were analyzed longitudinally in PSP target regions defined by the basal ganglia atlas,^28^ the Brainnetome atlas,^29^ and the Hammers atlas,^30^ in accordance with prior autopsy^19^ and in vivo PET data.^17^ Regions of interest (ROIs) included subcortical structures such as the internal and external part of the globus pallidus, putamen, subthalamic nucleus, substantia nigra, dorsal midbrain, and dentate nucleus, as well as cortical regions including the dorsolateral prefrontal cortex and medial prefrontal cortex. All analyses were performed both with and without partial volume effect correction (PVEC).^31^

### 2.5 Assessment of functional connectivity

To determine a functional connectivity template, we used resting‐state fMRI obtained on Siemens MRI scanners in 66 amyloid PET–negative cognitively normal subjects from the Alzheimer's Disease Neuroimaging Initiative (ADNI, 44 females). While it is conceivable that PSP‐related degeneration alters functional connectivity, particularly in regions affected by tau, using disease‐specific connectomes may obscure the propagation patterns we aim to study. In fact, once tau pathology has traversed and damaged a given connection, the resulting disconnection could mask prior pathways of spread. Therefore, modeling tau propagation on a healthy connectome likely better reflects the connectomic architecture that constrains the spatiotemporal evolution of pathology. Preprocessed fMRI data were obtained using our pre‐established processing pipeline including slice‐timing correction, motion correction and scrubbing, detrending, band‐pass filtering (0.01–0.08 Hz) detrending and nuisance regression (i.e., 6 motion regressors, white matter and CSF timeseries as well as time and dispersion derivatives), and spatial normalization to MNI space. Preprocessed fMRI data were used to determine functional connectivity for the Brainnetome atlas, defined as Fisher *z* transformed Pearson‐moment correlations of preprocessed fMRI timeseries between ROI pairs. Subject‐specific connectivity matrices were averaged to determine a connectivity template, negative values and autocorrelations were set to 0, density thresholded at 0.3, and distance transformed following our pre‐established approach.^32^

### 2.6 Assessment of covariance in tau PET change and individualized tau spreading ROIs

We assessed the inter‐regional covariance in tau accumulation rates, adopting our previously established approach.^33^, ^34^ Specifically, we determined the correlation of tau PET change rates (i.e. ΔVTr) between each possible Brainnetome ROI pair, resulting in a 246 × 246 matrix of covariance in tau accumulation. Within this [^18^F]PI‐2620 PET covariance matrix, autocorrelations were set to zero and all correlations were Fisher *z* transformed. Subject‐specific tau spreading ROIs were generated by grouping 95% of regions into 8 regions (i.e., octiles) according to their template‐based connectivity to 5% of brain regions with highest tau PET change rates (ΔVTr), that is, the subject‐specific tau epicenter (Q1 = strongest connectivity to the epicenter; Q8 = weakest connectivity to the epicenter).

### 2.7 Histological quantification of neurons in the pallidum

To quantify neurons in the pallidum, we performed histological analyses of hematoxylin and eosin (H&E) stains of 5 µm thick slices of formalin‐fixed and paraffin‐embedded tissue from coronal slices of the pallidum at the level of the mammillary bodies. Stains were digitized with a Zeiss Axio Scan Z.1 scanner with a magnification of 20 leading to a pixel size of 0.22 × 0.22 µm^2^, and further analyzed with Qupath (v0.6.0).^35^ First, the pallidum was manually segmented with the polygon tool. Second, the watershed cell detection tool was optimized by visually checking the annotations and adapting the parameters as follows to improve the detection of relatively large and sometimes dull appearing neurons compared to numerous smaller glial cells: “detectionImageBrightfield”: “Hematoxylin OD,” “requestedPixelSizeMicrons”: 1.0, “backgroundRadiusMicrons”: 12, “medianRadiusMicrons”: 0.0, “sigmaMicrons”: 2.0, “minAreaMicrons”: 10.0, “maxAreaMicrons”: 800.0, “threshold”: 0.1, “watershedPostProcess”: false, “cellExpansionMicrons”: 4.0, “includeNuclei”: true, “smoothBoundaries”: true, “makeMeasurements”: true. Third, a random forest object classifier was trained on these detected cells within Qupath based on three categories: neuronal, non‐neuronal (mostly glial and endothelial), and artifact. The latter mainly included small black formalin artifacts. In total, the object classifier was trained with 201 non‐neuronal cell labels, 168 neuronal cell labels, and 23 annotations of artifacts distributed over all 10 scans for a balanced performance. Subsequently, this algorithm was applied to the whole pallidum segmentations and neuron counts were extracted per mm^2^.

### 2.8 Statistics

Statistical analyses were conducted using R Studio (Version 4.3.1) and GraphPad Prism (Version 10). Group differences for demographic variables were tested using an unpaired *t* test for age and a chi‐square test for sex distribution. Paired *t* tests were used to assess longitudinal changes in [^18^F]PI‐2620 VTr values between baseline and follow‐up across predefined subcortical and cortical ROIs. To account for variability in follow‐up intervals (range 14–30 months), all longitudinal VTr changes were harmonized to the cohort's mean follow‐up duration (21.4 months) by linearly scaling individual ΔVTr values to this reference timepoint (ΔVTr harmonized = ΔVTr observed × 21.4 / individual follow‐up months). Similarly, clinical progression was assessed using paired *t* tests for PSPRS, UPDRS‐III, MoCA, and SEADL. To explore the relationship between baseline tau PET signals and subsequent signal change, a linear regression was computed between baseline VTr values and longitudinal ΔVTr (follow‐up minus baseline). In a second step, a multiple regression model included the baseline‐to‐follow‐up interval as covariate, followed by additional exploratory covariates (age, sex, disease duration, baseline PSPRS). Correlations between tau PET signal and clinical severity were assessed using Pearson correlations between tau PET *z* scores (normalized to healthy controls) and PSPRS scores at both timepoints, as well as for changes over time (ΔPSPRS). To test whether functionally connected brain regions show correlated tau accumulation rates, we assessed the association between the inter‐regional covariance in tau PET change rates and inter‐regional functional connectivity, both for the whole brain as well as constrained to subcortical ROIs of the Brainnetome atlas. In addition, we defined subject‐level tau accumulation epicenters as regions with the fastest tau accumulation over time. We then tested whether the seed‐based functional connectivity pattern of these tau epicenters predicted brain‐wide tau accumulation patterns (i.e., ΔVTr) on the subject level. To this end, we determined regression‐derived beta values of the association between epicenter connectivity and tau change rates and applied a one sample *t* test to these beta values. To assess differences between epicenters and octiles, we used a one‐way analysis of variance with Bonferroni multiple comparison post hoc test.

Last, we determined sample size estimates for tau‐targeting interventions with hypothetical intervention effects of 10%/20%/30%/40%/50%/75%/100% using G × Power 3.1.9.7 (settings: two‐sample *t* test, type I error rate *α* = 0.05, power = 0.8). In this regard, progression rates were normalized to an 18‐month interval in the internal part of the globus pallidus. The treatment effect was defined as the difference between the baseline‐adjusted follow‐up VTr (BL‐adjusted FUP‐PET) and a reduced follow‐up VTr reflecting the respective hypothetical intervention effect (e.g., 10% reduction of FUP‐PET). Effect sizes were calculated accordingly, based on the mean differences and baseline‐adjusted standard deviation of follow‐up values. All statistical tests were two tailed, and significance was defined as *P* < 0.05.

## 3 RESULTS

### 3.1 Demographics of the longitudinal in vivo PET imaging population

Of the 23 patients with PSP, 9 were clinically diagnosed with Richardson's syndrome (PSP‐RS) and 14 with non‐RS variants (PSP‐CBS, PSP with frontal presentation [PSP‐F], PSP with predominant Parkinsonism [PSP‐P], PSP with progressive gait freezing [PSP‐PGF]). The interval between baseline and follow‐up scans was 21.4  ±  4.3 months (range: 14–30 months). Demographics and clinical scores of the study cohort are reported in Table 1. The PSP patients did not differ significantly from the healthy controls (HCs) in terms of age (*P* = 0.57; minimum ages: PSP 55 years, HC 52 years) and sex (*χ* ^2^ = 2.45, *P* = 0.12), despite a higher proportion of males among PSP patients (74%).

### 3.2 Serial tau PET reveals progressive subcortical tracer accumulation in PSP

**FIGURE 1:**
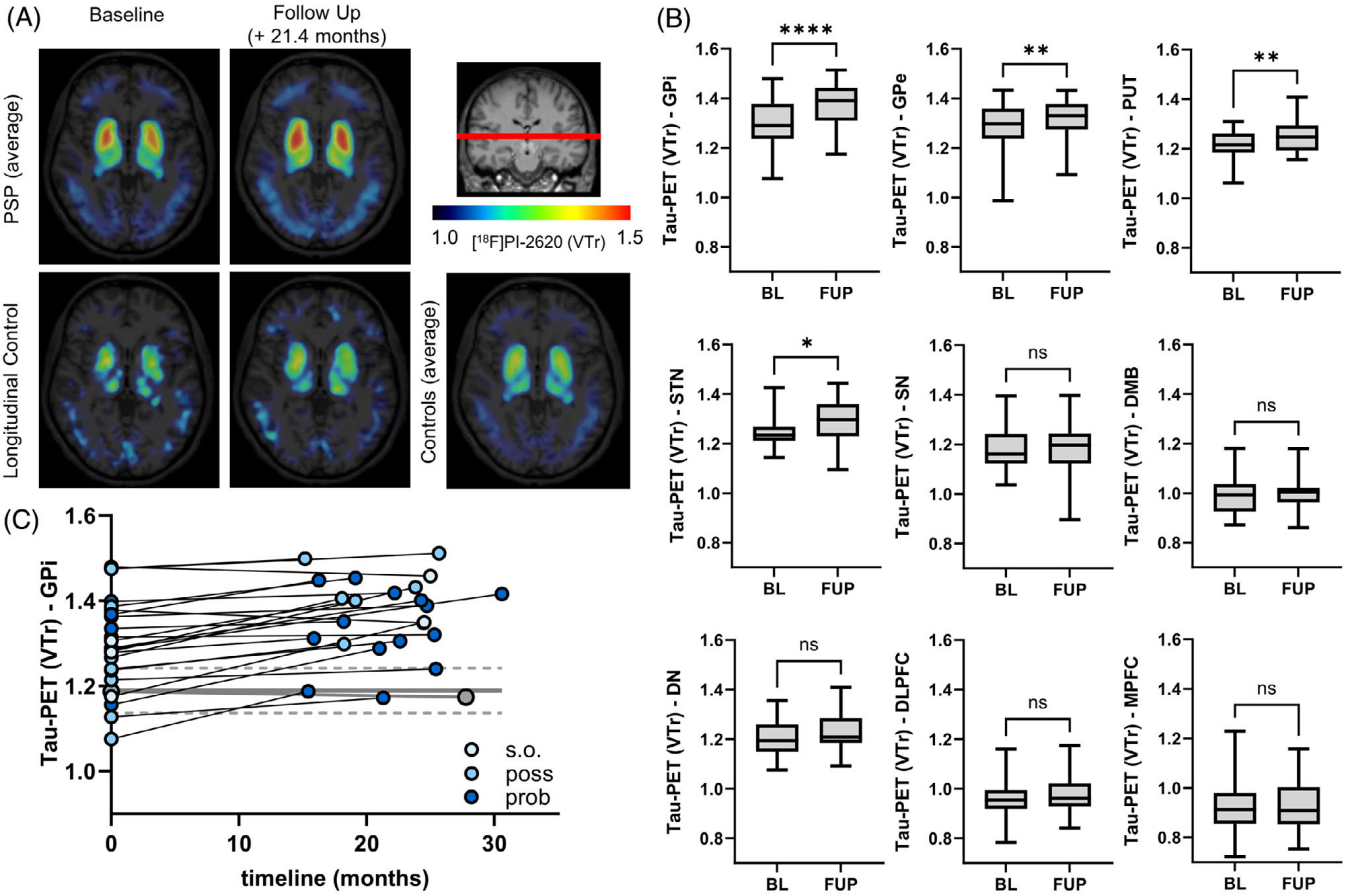
Longitudinal increases in subcortical tau positron emission tomography (PET) signal in patients with progressive supranuclear palsy (PSP). A, Axial [^18^F]PI‐2620 tau PET images at baseline (BL) and follow‐up (FUP) for patients with PSP (top row), the single healthy control subject with longitudinal imaging, and the cross‐sectional averaged healthy controls (bottom row) illustrate elevated tracer uptake in subcortical nuclei of PSP over time. B, Box plots show regional tau PET binding (volume of distribution ratio [VTr]) at BL and mean FUP (21.4 months) for multiple subcortical and cortical regions, including the internal part of the globus pallidus (GPi), external part of the globus pallidus (GPe), putamen (PUT), subthalamic nucleus (STN), substantia nigra (SN), dorsal midbrain (DMB), dentate nucleus (DN), dorsolateral prefrontal cortex (DLPFC), and medial prefrontal cortex (MPFC). Paired *t* tests were used to assess temporal differences in tau PET VTr. *P* values < 0.0332, 0.0021, 0.0002, and 0.0001 are shown as *, **, ***, and ****, respectively. C, Individual trajectories of GPi tau PET signal over time confirm a robust increase in most patients. Blue markers denote diagnostic certainty levels at baseline and follow‐up according to Movement Disorder Society Progressive Supranuclear Palsy criteria. The gray marker represents the single healthy control with longitudinal data. Gray dashed lines indicate the mean ± standard deviation of the healthy control group at baseline for reference.

[^18^F]PI‐2620 tau PET using a 4RT‐tailored temporo‐orbital reference region for intensity normalization revealed progressive tracer uptake in subcortical regions of patients with 4RT, as visually apparent in group‐averaged images from baseline to follow‐up compared to low uptake in healthy controls (Figure 1A, 2A). Quantitative analysis normalized to average follow‐up timepoint (21.4 months) confirmed significant longitudinal increases in tau PET binding (VTr) in subcortical regions, most prominently in the internal part of the globus pallidus (+5.5%; *P* < 0.0001), followed by the external part of the globus pallidus (+3.3%; *P* = 0.0013), putamen (+2.6%; *P* = 0.0071), and subthalamic nucleus (+3.3%; *P* = 0.041). No significant changes were observed in the substantia nigra (+1.1%; *P* = 0.66), dorsal midbrain (+1.2%; *P* = 0.38), dentate nucleus (+1.5%; *P* = 0.10), dorsolateral prefrontal cortex (+2.0%; *P* = 0.11), or medial prefrontal cortex (+1.6%; *P* = 0.32; Figure 1B). Individual tau PET trajectories revealed consistently increasing signal in the basal ganglia, most prominently in the internal part of the globus pallidus, across most patients, while the single HC remained within the reference range (Figure 1C). These results remained consistent after applying PVEC (Figure S1 in supporting information). In contrast, when using a cerebellar reference region, longitudinal differences between baseline and follow‐up largely disappeared (Figure S2 in supporting information).

**FIGURE 2:**
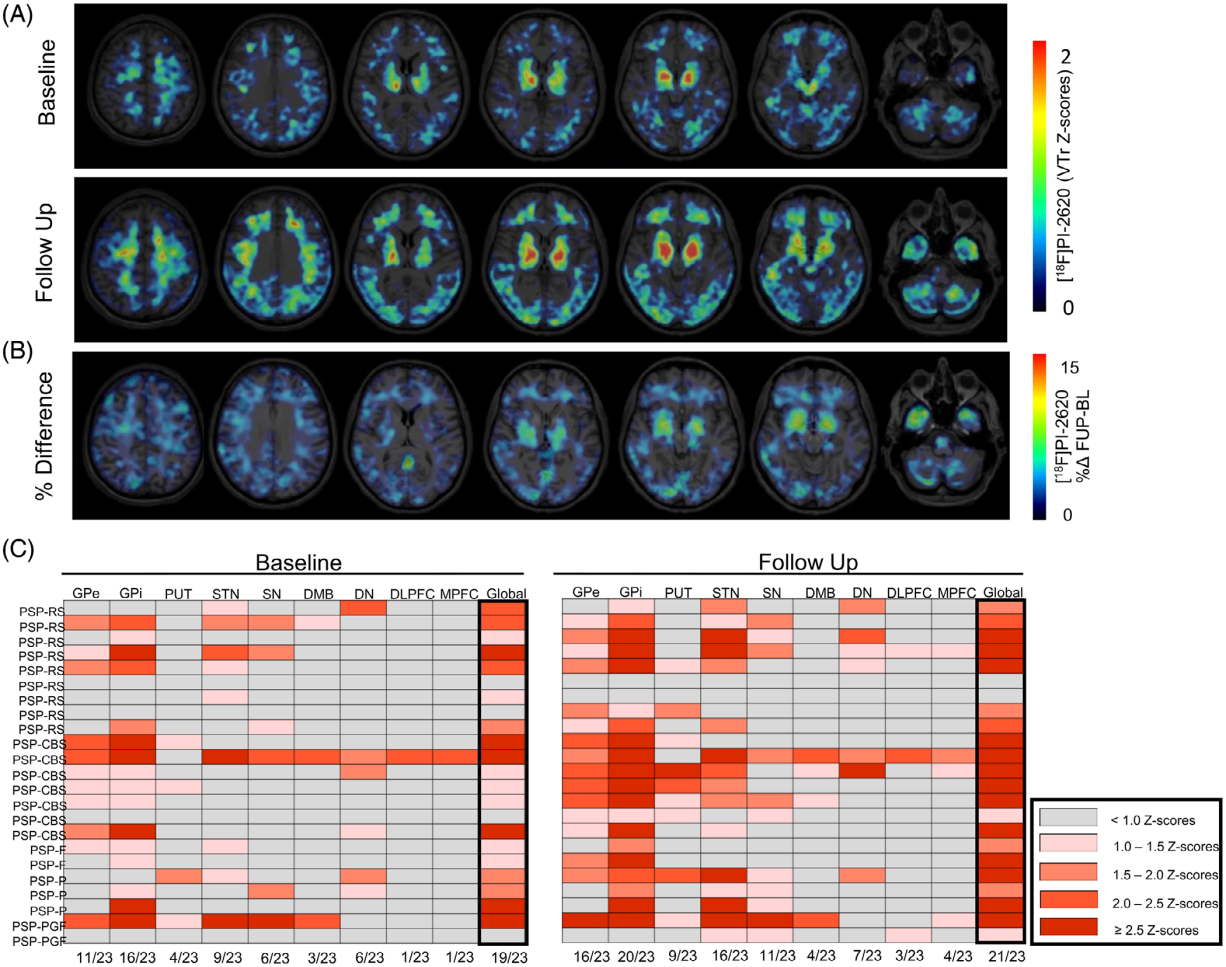
Individual‐level progression of tau positron emission tomography (PET) signals in patients with progressive supranuclear palsy (PSP). A, Averaged axial [^1^ ^8^F]PI‐2620 tau PET images at baseline (BL) and follow‐up (FUP) illustrate progressive subcortical and cortical tracer uptake. Images are shown as volume of distribution ratio (VTr) *z* score maps normalized to the healthy control group. B, Axial [^18^F]PI‐2620 tau PET images showing the percentage difference (%Δ FUP – BL), highlighting spatial patterns of longitudinal change across the brain. C, Heatmaps show the distribution of elevated tau PET signal (*z* scores) across subcortical and cortical regions at baseline (left) and follow‐up (right) for each individual. Colors indicate the degree of deviation from the healthy control mean in *z* scores. Numbers at the bottom indicate how many individuals exceeded the 1 standard deviation threshold in each region. DLPFC, dorsolateral prefrontal cortex; DMB, dorsal midbrain; DN, dentate nucleus; GPe, external part of the globus pallidus; GPi, globus pallidus; MPFC, medial prefrontal cortex; PSP‐CBS, progressive supranuclear palsy with corticobasal syndrome; PSP‐F, progressive supranuclear palsy with frontal presentation; PSP‐P, progressive supranuclear palsy with predominant Parkinsonism; PSP‐PGF, progressive supranuclear palsy with progressive gait freezing; SN, substantia nigra; STN, subthalamic nucleus.

To complement the regional analyses, we additionally generated whole‐brain voxel‐wise percentage‐difference maps (Figure 2B). These exploratory maps provide a spatial overview of longitudinal tau PET change across the cortex and subcortex. While subcortical regions showed the most pronounced changes, cortical percentage differences were smaller and spatially heterogeneous across individuals, consistent with the heterogeneous phenotype composition of the cohort.

Because the internal globus pallidus showed the strongest and most reliable longitudinal change in our dataset, and has repeatedly been identified as a key region for detecting 4R tau pathology in prior in vivo and *post mortem* studies, we selected this region for subsequent sensitivity and power analyses.^17^, ^19^, ^36^, ^37^ To assess the robustness of these findings and the sensitivity of [^18^F]PI‐2620 tau PET for detecting longitudinal change, we conducted a post hoc power analysis based on the normalized 18‐month progression rate in the internal part of the globus pallidus (mean ΔVTr  = 0.058, standard deviation [SD] = 0.048). Using an α level of 0.05 and the corresponding effect size (Cohen d) of 0.571, this analysis yielded a statistical power of 94.3% with a required sample size of *n* = 15 to achieve > 80% power. To further inform future clinical trial designs, we additionally estimated the sample sizes required to detect hypothetical tau‐targeting intervention effects ranging from 10% to 100% reduction in longitudinal tau accumulation. Based on the baseline‐adjusted variability of follow‐up VTr and scaled effect sizes for different intervention magnitudes, the estimated sample sizes required per group (*α* = 0.05, power = 80%) were *n* = 1257 (10% reduction), *n* = 316 (20%), *n* = 137 (30%), *n* = 76 (40%), *n* = 47 (50%), *n* = 19 (75%), *n* = 9 (100%).

Individual‐level analysis based on *z* score normalization relative to the healthy control group further illustrated progression patterns. To sensitively capture early or subtle regional involvement in this heterogeneous PSP cohort, we applied a threshold of ≥ 1 SD above the healthy control mean to indicate elevated tau PET signal. At baseline, 19 out of 23 patients (83%) showed elevated tau PET signal in at least one subcortical region, most frequently in the globus pallidus. By follow‐up, this number increased to 21 out of 23 patients (91%), with additional and stronger involvement of the putamen, subthalamic nucleus, and substantia nigra. Region‐specific comparisons revealed marked increases in the number of affected individuals: internal part of the globus pallidus: from 16 (70%) to 20 (87%), external part of the globus pallidus: from 11 (48%) to 16 (70%), putamen: from 4 (17%) to 9 (39%), and subthalamic nucleus: from 9 (39%) to 16 (70%; Figure 2C).

### 3.3 Serial tau PET reveals a ceiling effect of tau pathology in PSP

**FIGURE 3:**
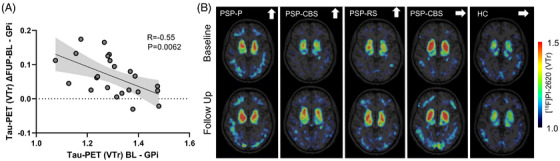
Baseline tau burden predicts longitudinal change in tau positron emission tomography (PET) signals of the basal ganglia. A, Linear regression model depicts negative association between baseline GPi tau PET signal (volume of distribution ratio [VTr]) and change over time (ΔVTr, follow‐up minus baseline) in progressive supranuclear palsy (PSP). Shaded area indicates 95% confidence interval. B, Representative axial [^18^F]PI‐2620 tau PET images at baseline and follow‐up from individuals with PSP‐P, PSP‐CBS, and PSP‐RS, compared to a healthy control (HC). Arrows indicate increasing (↑) or stable (→) GPi signal over time. GPi, globus pallidus; PSP‐CBS, progressive supranuclear palsy with corticobasal syndrome; PSP‐P, progressive supranuclear palsy with predominant Parkinsonism; PSP‐RS, progressive supranuclear palsy with Richardson's syndrome.

Having identified the internal part of the globus pallidus as the region with the most pronounced longitudinal increase in tau PET signal, we next examined whether baseline tau burden in this region predicted subsequent accumulation. To this end, we assessed the relationship between baseline tau PET signal in the pallidum and longitudinal change across individuals with PSP. A significant negative association was observed between baseline tau PET signal and longitudinal tau PET change (*R* = –0.55, *P* = 0.0062), indicating that individuals with lower baseline signal showed greater subsequent increases in tau PET (Figure 3A). When adjusting for scan interval in a multiple regression model, baseline tau PET remained a significant predictor (*β* = –0.55, *P* = 0.008). The overall model was significant (*R* ^2^ = 0.31, *F*[2,20] = 4.51, *P* = 0.024), while the scan interval itself did not contribute significantly (*β* = –0.067, *P* = 0.72). Furthermore, inclusion of additional covariates, including disease duration (*β* = 0.00, *P* = 0.78), sex (*β* = –0.024, *P* = 0.42), age (*β* = 0.00, *P* = 0.94), and baseline clinical impairment as measured by the PSP rating scale (*β* = –0.00, *P* = 0.38), did not improve model fit, indicating that none of these factors significantly predicted longitudinal tau PET change.

This relationship remained consistent after PVEC, suggesting that this effect was not driven by atrophy (Figure S3B in supporting information). Representative individual tau PET images supported this finding: patients with initially low signal in the pallidum showed marked increases over time, whereas individuals with already elevated baseline values remained stable or showed only minor changes. In contrast, the HC subject demonstrated low and stable tracer uptake across serial timepoints, which remained consistent after PVEC (Figure 3B and Figure S4 in supporting information). Taken together, our data suggest a potential ceiling effect of tau accumulation in subcortical regions, where advanced baseline pathology may limit further detectable tracer increases.

### 3.4 Clinical progression and its relationship to subcortical tau PET signals

**FIGURE 4:**
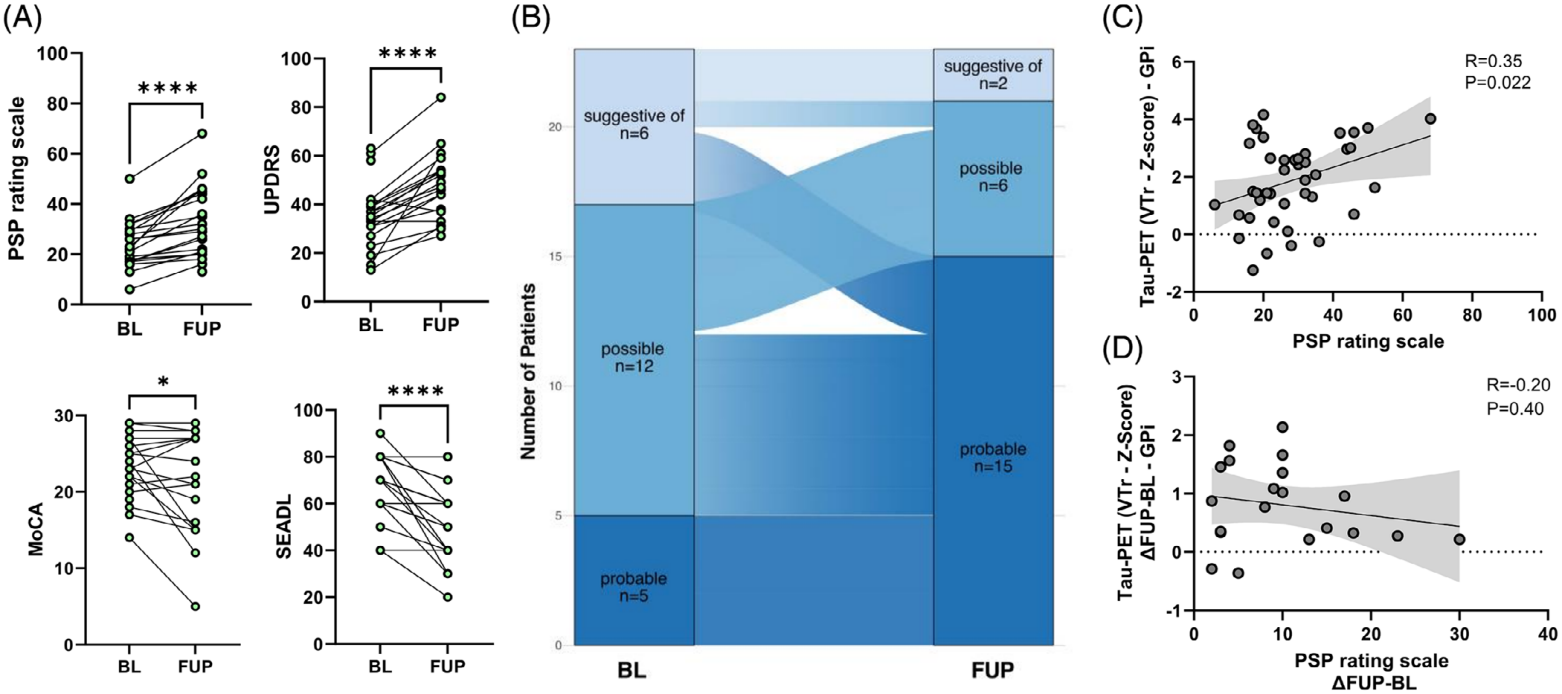
Clinical progression and its relationship to tau positron emission tomography (PET) signal in progressive supranuclear palsy (PSP). A, Line plots showing individual clinical scores at baseline (BL) and follow‐up (FUP) for the PSP Rating Scale, UPDRS‐III, Montreal Cognitive Assessment (MoCA), and Schwab and England Activities of Daily Living scale (SEADL). B, The alluvial chart illustrates diagnostic transitions over time, showing how individuals progressed from “suggestive of” (s.o.) or possible PSP to probable PSP, or remained as a probable PSP at follow‐up according to Movement Disorder Society criteria. C, Scatter plot depicts globus pallidus (GPi) tau PET *z* scores at BL and FUP plotted against respective PSP Rating Scale scores at the same timepoint. D, Scatter plot shows the change in GPi tau PET *z* scores from baseline to follow‐up (ΔFUP – BL) in relation to the corresponding change in PSP Rating Scale scores.

Across the observation period, patients with PSP showed clear clinical deterioration. Individual trajectories revealed significant increases in disease severity as measured by the PSPRS (+47.9%, *P* < 0.0001) and UPDRS‐III (+57.3%, *P* < 0.0001), alongside a decline in activities of daily living (SEADL scale: –24.6% *P* < 0.0001) and cognitive performance (MoCA: –11.3%, *P* = 0.048; Figure 4A). Diagnostic certainty according to MDS criteria also reflected this clinical progression: at baseline, patients were distributed across “suggestive of” (s.o.; *n* = 6), “possible” (poss, *n* = 12), and “probable” (prob, *n* = 5) PSP categories.^13^ By follow‐up, a larger proportion of individuals (s.o.: 67%; poss: 58%) had transitioned to a category of higher diagnostic certainty, while fewer remained in earlier stages (s.o.: *n* = 2; poss: *n* = 6; prob: *n* = 15; Figure 4B). Tau PET findings did not contribute to the assignment of diagnostic certainty categories. There were no changes to a non‐PSP diagnosis at follow‐up. We next examined the relationship between clinical severity and tau PET signal. When pooling both baseline and follow‐up tau PET *z* scores in the internal part of the globus pallidus, a significant positive cross‐sectional correlation with PSPRS scores was observed (*R* = 0.35, *P* = 0.022), which remained consistent after PVEC (Figure 4C and Figure S3C). However, no significant correlation was observed between longitudinal changes in tau PET signals and clinical progression as measured by the change in PSPRS scores over time (*R* = –0.20, *P* = 0.40; Figure 4D and Figure S3D). Although baseline tau PET tended to be slightly lower in individuals that progressed in diagnostic certainty stages from those who did not, no significant statistical differences were observed between both groups (*P* = 0.21; Figure S5A in supporting information). Similarly, both baseline tau PET (area under the curve [AUC] = 0.62, *P* = 0.32) and tau PET change (AUC = 0.61, *P* = 0.36) did not discriminate in regard to diagnostic certainty progression (Figure S5B, C).

### 3.5 Functional connectivity between brain regions determines longitudinal change rates of tau pathology

**FIGURE 5:**
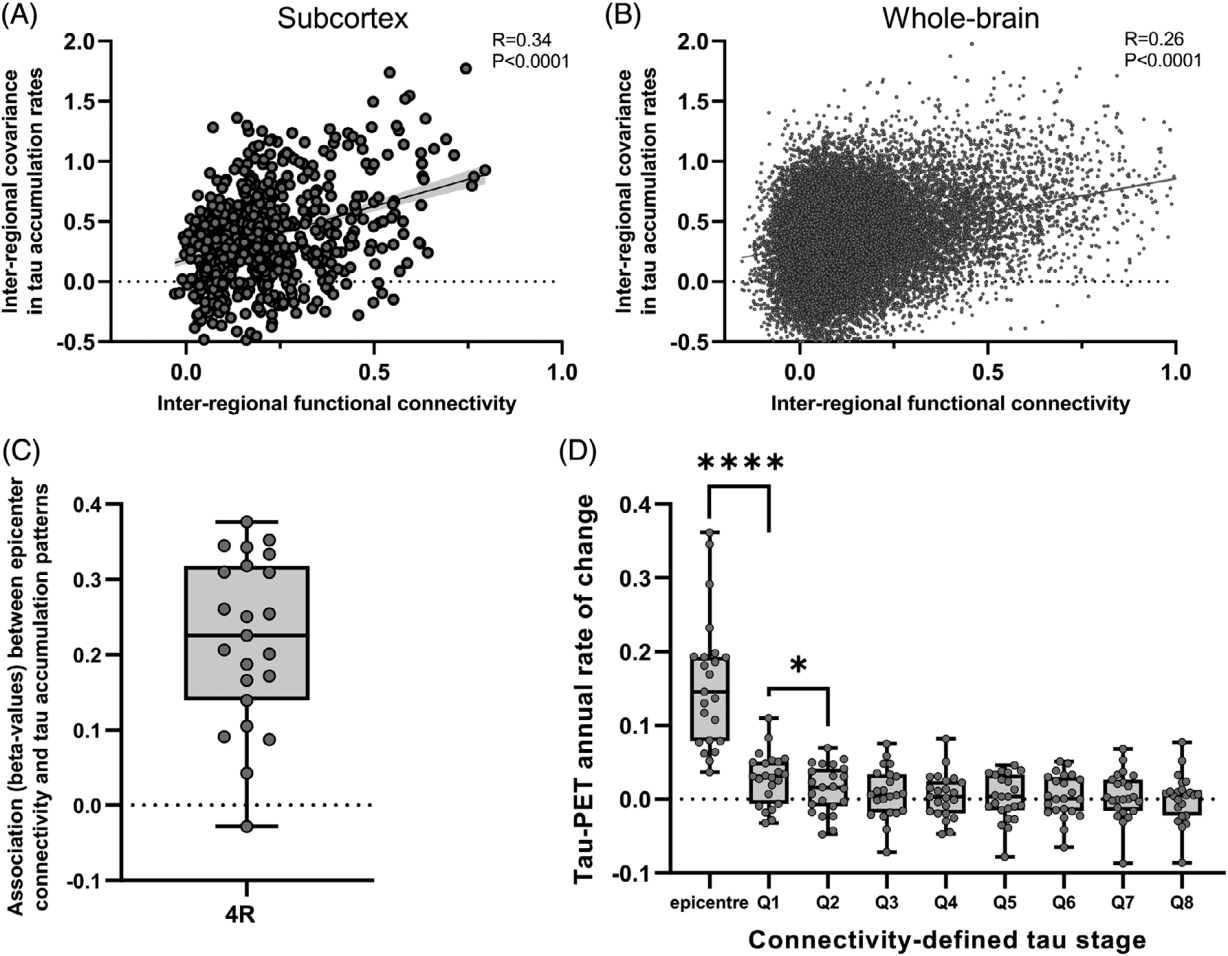
Association between functional connectivity and covariance in longitudinal tau positron emission tomography (PET) change. A, B, Scatter plot depicts the positive association between inter‐regional functional connectivity and covariance in tau PET accumulation rates at the subcortex (A) and whole‐brain (B) level. Shaded area indicates 95% confidence interval. C, Boxplot depicts the association between connectivity to tau epicenters (defined as the 5% of regions with fastest tau accumulation per subject) and brain‐wide tau PET accumulation. D, Boxplots depict a connectivity‐defined gradient of tau PET accumulation rates across epicenters and octiles (Q1–Q8) of decreasing connectivity to epicenters. Boxplots are displayed as median (center line) ± interquartile range (box boundaries) with whiskers. A one‐way analysis of variance with Bonferroni post hoc tests was used to assess differences between epicenters and octiles. *P* values < 0.0332 to 0.0001 are indicated as * and **, corresponding to the differences between Q1 and Q2, and between the epicenter and Q1, respectively.

Further, we tested whether functionally connected brain regions show correlated tau PET change rates (i.e., ΔVTr) to determine whether the brains functional network architecture constrains tau accumulation and spread, in line with the trans‐neuronal spreading hypothesis of tau pathology. Supporting this, we found that strongly functionally connected regions indeed showed stronger covariance in tau accumulation rates, while less connected regions did not, both for the subcortex (*R* = 0.34, *P* < 0.0001) as well as for the whole brain (*R* = 0.26, *P* < 0.0001; Figure 5A, B). Next, we determined whether subject‐level tau accumulation rates followed the connectivity pattern of tau epicenters, defined as 5% of brain regions with fastest tau accumulation in each patient. Here, we found that the association between epicenter connectivity and brain‐wide tau accumulation rates was significantly positive (*P* < 0.0001) across all patients, where regions with closest connectivity to tau accumulation epicenters showed fastest tau accumulation, whereas regions less connected to tau epicenters showed slower tau accumulation rates (Figure 5C). This was also reflected in a gradient of tau accumulation from tau accumulation epicenters across brain regions divided into eight subregions (i.e., octiles), depending on their connectivity strength to tau accumulation epicenters. Here, regions most closely connected to tau accumulation epicenters (i.e., Q1) showed fastest tau accumulation rates, while tau accumulation decreased gradually, with further connectivity‐based distance to these epicenters (i.e. Q2–Q8; Figure 5D). Epicenters differed significantly from all octiles (all *P* < 0.0001). Furthermore, Q1 differed significantly from every subsequent octile (e.g., Q1 vs. Q2: *P* = 0.0113), demonstrating a robust connectivity‐defined gradient. Together, these results suggest that highly connected brain regions show similar tau accumulation rates, and that tau propagates from fast accumulating epicenters across connected brain regions.

### 3.6 Neuronal loss contributes to the ceiling effect of tau PET signal in PSP

**FIGURE 6:**
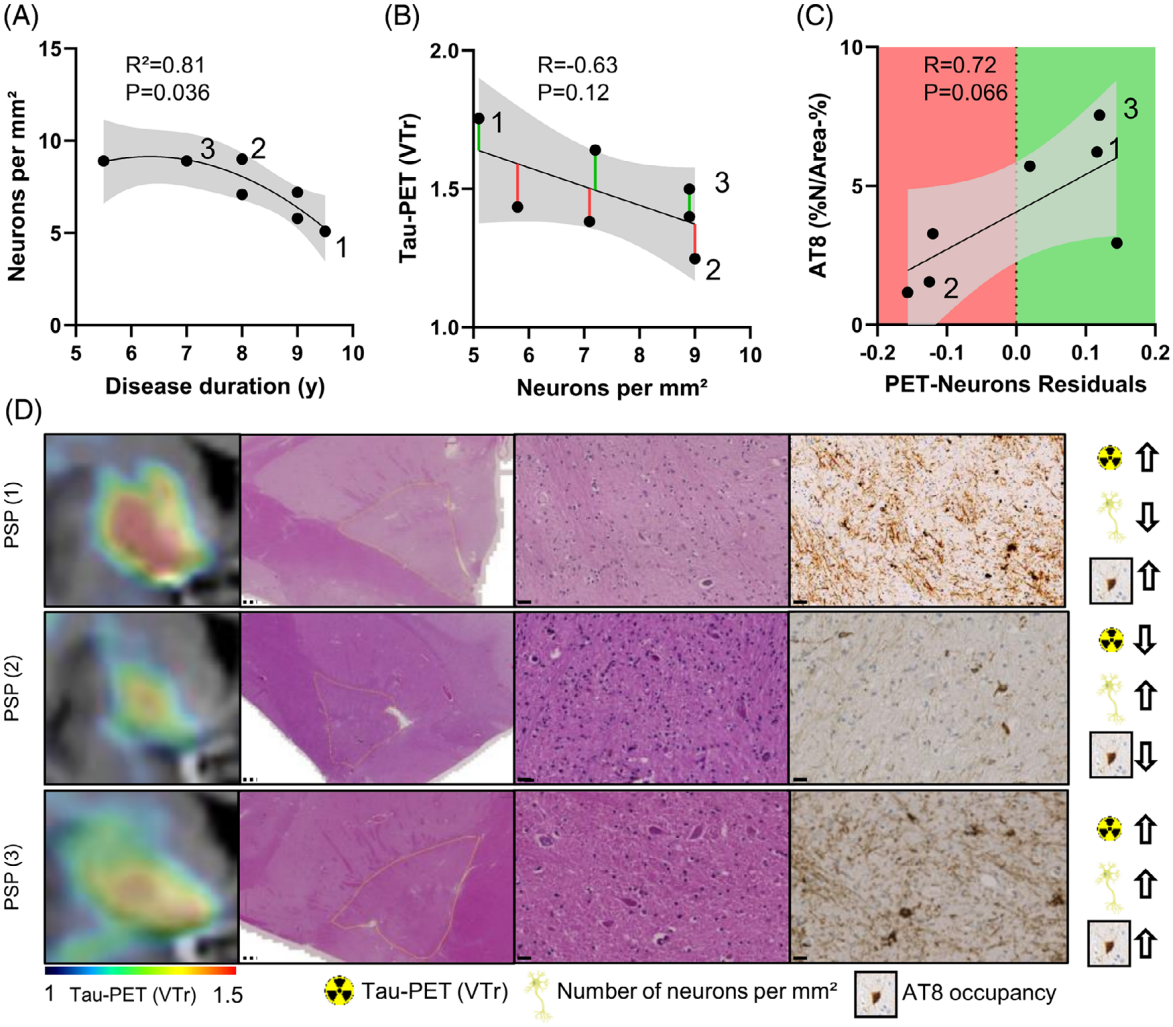
Relationship between tau positron emission tomography (PET) signal and neuronal density in the globus pallidus. A, Non‐linear quadratic regression of neuronal density (cells/mm^2^) and disease duration. B, C, Residuals from the linear regression between tau PET and neuronal density are color‐coded as positive (green) or negative (red) and plotted against AT8 positive area. Linear regression lines (including 95% confidence intervals) were calculated in samples derived from seven patients with definite progressive supranuclear palsy (PSP).^19^ D, Representative coronal [^18^F]PI‐2620 tau PET images of the globus pallidus and corresponding hematoxylin and eosin (H&E) as well as AT8 stained sections are shown for three PSP cases. White arrows summarize the observed directionality between tau PET signal intensity, neuronal abundance, and AT8 occupancy. Scale bars: overview image = 1 mm (dotted line); high‐magnification image = 20 µm (straight line). *R* indicates Pearson correlation coefficient. VTr, volume of distribution ratio.

As an exploratory analysis, we investigated whether loss of neuronal density impacts the ceiling effect observed in longitudinal tau PET. To this end, we reanalyzed autopsy samples of our previously published, independent cohort consisting of seven patients with pathologically confirmed PSP, who were not part of the longitudinal imaging cohort, and quantified neuronal density and corresponding tau PET signal in the globus pallidus.^19^ A non‐linear (quadratic) regression best described the relationship between neuronal density and disease duration, indicating the neuronal loss upon disease progression (*R* ^2^ = 0.81, *P* = 0.036; Figure 6A). Furthermore, neuronal density tended to decrease with higher tau PET signal (*R* = –0.63, *P* = 0.12), though this association was not statistically significant (Figure 6B). To assess whether elevated PET signal exceeding predictions based on neuronal density reflects disproportionate tau pathology, we calculated residuals from the tau PET–neuron density regression. These residuals were then related to regional AT8 immunoreactivity as a measure of tissue tau burden. Cases with positive residuals (i.e., higher PET signal than predicted based on neuronal density) showed a tendency toward elevated AT8 occupancy (*R* = 0.72, *P* = 0.066; Figure 6C), suggesting that tau PET might be able to capture high tau burden in the globus pallidus even in the presence of advanced neuronal loss (Figure 6C, D).

## 4 DISCUSSION

We provide the first comprehensive in vivo longitudinal assessment of PSP using next‐generation [^18^F]PI‐2620 tau PET. Serial tau PET revealed progressive non‐linear increase of subcortical tracer signal over ≈ 21 months, in which individuals with low baseline tau PET demonstrated greatest longitudinal increases, whereas those with high baseline signals exhibited minimal or no accumulation. This suggests a saturation or “ceiling effect” of tracer binding once dense tau aggregates are established, consistent with observations of longitudinal tau PET in AD, in which early‐involved regions show a saturation as they accumulate dense NFTs and undergo advanced neurodegeneration.^38^, ^39^

Although both PSP and AD can exhibit a plateauing of tau PET at high tau levels, the underlying neuropathological composition differs markedly between diseases. In AD, ghost tangles may persist after neuronal death and show residual tau‐tracer binding, although weaker and more variable than intraneuronal paired helical filament tau.^40^, ^41^, ^42^, ^43^ In contrast, PSP typically lacks ghost tangles, and tau aggregates appear to be cleared more rapidly once neurons degenerate.^44^ While PSP brains do develop NFTs in neurons, very few reach the “ghost tangle” stage with virtual absence in subcortical target areas such as the pallidum or midbrain.^45^ As a result, tau PET in PSP likely captures ongoing intracellular tau accumulation, with little or no additional ghost‐tangle signal once tau pathology has saturated. Although astrocytic and oligodendroglial tau inclusions are also prominent in 4RT, we recently reported that the in vivo tau PET signal is primarily driven by neuronal tau aggregates.^19^ This pathological PSP versus AD difference also translates into distinct clinical–imaging relationships. Longitudinal tau PET studies in AD consistently showed that cortical tau accumulation closely tracks cognitive decline,^46^ and a large cohort study demonstrated that tau PET change predicts future cognitive deterioration across the AD spectrum.^47^ Together, these factors explain why tau PET in PSP may plateau despite ongoing clinical decline, and why the ceiling effect appears earlier, more globally, and more definitively in 4RT than in AD.

While tau PET changes were most pronounced in the pallidum and surrounding basal ganglia structures, cortical regions showed only modest changes across individuals, consistent with the known distribution of tau pathology 4RT.^17^, ^48^ Importantly, cortical involvement in PSP is known to vary across clinical subtypes: PSP‐CBS typically shows a more pronounced cortical tau involvement,^48^, ^49^ whereas PSP‐F demonstrates selective frontal involvement compared to PSP‐RS.^50^ In light of this subtype‐specific heterogeneity and the limited sample size of our phenotypically mixed cohort, the absence of statistically significant cortical increases in our data should not be interpreted as evidence of true cortical stability.

Despite this, clinical deterioration was evident across our entire PSP cohort. Notably, longitudinal tau PET changes did not correlate with clinical progression, suggesting a dissociation between pathological burden and functional decline, aligning with the concept of a ceiling effect: once regional tau burden reaches a pathological plateau, further neuronal dysfunction and clinical worsening may proceed independently of PET‐detectable tau changes. In this context, a stable tau PET signal does not necessarily indicate clinical stability, particularly in advanced disease stages in which tau pathology may have saturated vulnerable regions.

A similar interpretation was raised in a prior longitudinal study using [^18^F]flortaucipir in PSP, which reported only modest 1‐year signal increases, yet, [^18^F]flortaucipir may not reliably quantify 4R tau. Therefore, [^18^F]PI‐2620 offers several advantages for imaging 4RT, in which preclinical, *post mortem*, and in vivo studies have demonstrated higher 4R tau binding affinity, with minimal off‐target binding in the basal ganglia.^19^, ^26^ As [^1^ ^8^F]PI‐2620 binds both 4R and mixed 3/4R tau aggregates, it enables the detection of frequent co‐pathologies, in which concomitant AD‐type tau is present in a substantial proportion of 4RT cases.^14^, ^48^, ^51^ Disease‐specific regional binding patterns, binding strength, and tracer kinetics nevertheless allow differentiation between underlying tau pathologies despite overlapping isoforms.^16^, ^17^, ^52^ Together, these properties enhance the specificity and reliability of [^18^F]PI‐2620 for quantifying disease‐related tau changes in 4RT, supporting its use for longitudinal assessments and potential therapeutic monitoring, while future purely 4R‐selective tracers may further refine isoform‐specific imaging.

Other tracers such as [^18^F]florzolotau have shown promising cross‐sectional detection of 4R‐like binding patterns in the midbrain and pallidum while accurately distinguishing 4RT from other parkinsonian syndromes.^53^, ^54^, ^55^ However, longitudinal data for [^18^F]florzolotau or other 4R‐sensitive tracers are currently lacking, hence temporal dynamics of 4R tau accumulation cannot yet be compared across tracers.

To explore the ceiling effect in longitudinal tau PET, we investigated whether neuronal loss might influence tracer signals in PSP. Our autopsy analysis revealed a non‐linear relationship between disease duration and neuronal density, consistent with progressive cell loss.^56^ In parallel, we observed negative associations between neuronal density and tau PET, paradoxically suggesting that regions with fewer surviving neurons still show elevated tau PET uptake. This counterintuitive finding likely reflects disproportionate tau accumulation in remaining neurons in advanced disease. In such cases, a small number of tau‐laden neurons may continue generating strong PET signals.^19^ Supporting this, residuals from the tau PET–neuron density regression were positively associated with AT8 burden, indicating that PET signals remain high when remaining neurons disproportionately accumulate tau. Together, these findings suggest that the plateau in tau PET signals in PSP may not reflect the plateau of tau accumulation, but rather a shift in its cellular distribution, in which ongoing pathology is concentrated in fewer surviving neurons. This interpretation also aligns with recent multimodal imaging findings showing that [^18^F]PI‐2620 tau PET provides diagnostic information beyond structural and volumetric MRI, thereby improving overall diagnostic accuracy in PSP.^57^

We further demonstrate that tau PET accumulation is significantly correlated between functionally connected brain regions, supporting the view that tau propagates along brain networks thereby extending the network‐based tau spreading concept established in AD to 4RT.^33^, ^34^ This complements recent cross‐sectional work demonstrating that 4R tau PET signals and *post mortem* tau pathology covary across functionally connected brain regions in PSP.^33^, ^58^ Our results extend this concept by showing that functional connectivity not only shapes spatial tau distribution, but also constrains tau accumulation over time. Moreover, our patient‐centered epicenter‐based approach further supports that regions closely connected to tau epicenters accumulate tau more rapidly.

Our analyses used a temporo‐orbital white matter reference, previously shown to enhance the clinical interpretability of [^18^F]PI‐2620 PET signals in 4RT.^25^ This region lies within deep temporo‐orbital white matter, which consistently showed minimal 4R tau burden in neuropathological studies and negligible [^18^F]PI‐2620 binding in our prior PET‐to‐autopsy work.^19^, ^25^ Because white matter tau pathology in PSP is largely confined to subcortical fiber tracts and gray matter/white matter boundary zones,^19^, ^56^, ^59^ using a deep white matter reference region minimizes the risk of including tau‐affected tissue and provides greater longitudinal stability than cerebellar normalization.^25^ In contrast, the use of a cerebellar reference has been associated with reduced sensitivity to detect meaningful group differences, particularly in subcortical target regions.^60^ This limitation likely stems from lower target‐to‐reference contrast due to a combination of off‐target binding in the vermis and on‐target uptake in the dentate nucleus, both of which compromise its suitability as a stable reference region in 4RT.^27^ Such vermal off‐target retention has been reported across multiple tau tracers, including [^18^F]AV‐1451,^40^ [^18^F]RO‐948,^61^ [^18^F]MK‐6240,^62^ and [^18^F]PI‐2620,^27^ and is thought to arise from binding to neuromelanin‐containing leptomeningeal cells, analogous to the well‐known neuromelanin‐driven off‐target signal in the substantia nigra.^63^, ^64^ Nevertheless, we acknowledge that cross‐ligand and multi‐site validation of the novel temporo‐orbital white matter reference remains an important future step.

One strength of our study is the inclusion of various PSP subtypes, which may improve the generalizability of our findings to other PSP populations. Our subtype distribution (39% PSP‐RS) reflects the clinical heterogeneity of PSP and is broadly compatible with a large multi‐center autopsy‐confirmed study which reported ≈ 24% PSP‐RS among 100 cases.^65^ However, larger studies need to determine whether the observed PET signal changes differ meaningfully across the clinical variants of PSP.

Another strength of our study lies in the use of dynamic tau PET and blood flow–corrected IDIF‐based quantification, enabling the generation of VTr maps that are less susceptible than static measures to be confounded by regional perfusion changes. In addition to improving quantification, the dynamic acquisition protocol allows for parametric imaging, which facilitates high‐resolution visual interpretation of tracer distribution.^36^ This combination of kinetic modeling and standardized visual interpretation enhances both sensitivity and reader confidence in clinical assessment.

Nonetheless, certain limitations should be acknowledged. Most notably, the relatively small sample size and the inclusion of only a single HC with longitudinal tau PET restrict the generalizability of the results. Although tau accumulation can occur in healthy individuals over time, we considered this effect to be negligible, as supported by previous research.^5^ Second, we did not observe relevant longitudinal tau PET changes in the cortex; whether this reflects true absence of pathology progression or limitations of sensitivity in cortical areas remains to be explored. Third, while we found robust group‐level increases in subcortical tau PET signal, we did not observe a significant correlation between tau PET changes and clinical deterioration across individuals.

Overall, we provide first in vivo evidence that second‐generation [^18^F]PI‐2620 tau PET can successfully track progressive subcortical tau accumulation in primary 4R tauopathies. We demonstrate a non‐linear trajectory of tau accumulation, with a saturation effect in regions of high baseline pathology, and a dissociation from clinical progression. [^18^F]PI‐2620 offers high specificity and subcortical target contrast, particularly when combined with dynamic acquisition and IDIF‐based quantification.

## CONFLICT OF INTEREST STATEMENT

A.Z. has received: speaker honoraria from Dr. Willmar Schwabe GmbH, Pfizer, AstraZeneca; research support from Dr. Willmar Schwabe GmbH. M.B. is a member of the Neuroimaging Committee of the EANM. M.B. has received speaker honoraria from Roche, GE Healthcare, Iba, and Life Molecular Imaging; has advised Life Molecular Imaging and GE Healthcare; and is currently on the advisory board of MIAC. N.F. received speaker or consulting honoraria from Life Molecular Imaging, MSD, GE Healthcare, Eisai, and Biogen. R.A.W. has received speaker honoraria from Novartis/AAA and PentixaPharm and reports advisory board work for Novartis/AAA and Bayer. J.L. reports speaker fees from Bayer Vital, Biogen, EISAI, Lilly, TEVA, Bial, Zambon, Esteve, Merck, and Roche; consulting fees from Axon Neuroscience, EISAI, Alnylam, and Biogen; author fees from Thieme medical publishers and W. Kohlhammer GmbH medical publishers; and is an inventor in a patent “Oral Phenylbutyrate for Treatment of Human 4‐Repeat Tauopathies” (PCT/EP2024/053388) filed by LMU Munich. In addition, he reports compensation for serving as chief medical officer for MODAG GmbH, is beneficiary of the phantom share program of MODAG GmbH, and is inventor in a patent “Pharmaceutical Composition and Methods of Use” (EP 22 159 408.8) filed by MODAG GmbH, all MODAG activities outside the submitted work. The remaining authors declare that they have no conflicts of interest relevant to this study. Author disclosures are available in the supporting information.

## CONSENT STATEMENT

All participants (or their legal representatives) provided a written consent for PET imaging. The study protocol and PET data analyses were approved by the local ethics committee (LMU Munich, application numbers 17‐569 and 19‐022).

## Supporting information



Supporting Information

Supporting Information
