# Supplementary material for: Longitudinal monitoring of tau aggregation in progressive supranuclear palsy with [18F]PI‐2620 PET
Source: Alzheimers Dement. 2026 Feb 24;22(2):e71195. doi: 10.1002/alz.71195 (PMC12932912; doi:10.1002/alz.71195)
Supplement: Supplementary file 1 — Supporting Information [file ALZ-22-e71195-s002.docx]

**Supplementary Material to:**

**Longitudinal monitoring of tau aggregation in progressive supranuclear palsy with [^18^F]PI-2620 PET**

Contains Supplementary Figures:

- Supplementary Figure 1
- Supplementary Figure 2
- Supplementary Figure 3
- Supplementary Figure 4
- Supplementary Figure 5

**Supplementary Figure 1**


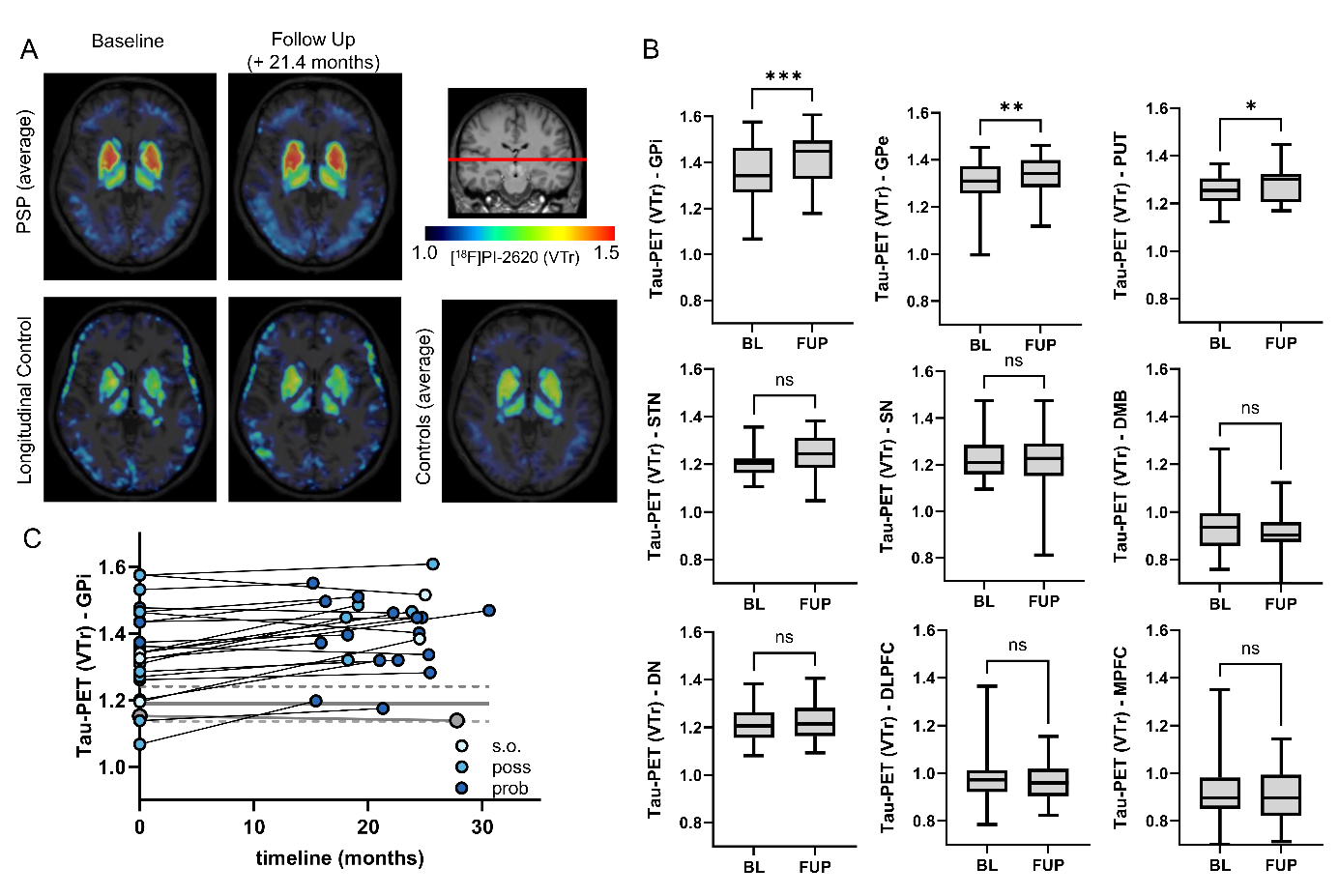


**Supplementary Figure 1**. **Longitudinal increases in subcortical tau-PET signal in patients with PSP after partial volume effect correction.** (**A**) Axial [^18^F]PI-2620 tau-PET images at baseline (BL) and follow-up (FUP) for patients with PSP (top row), the single healthy control subject with longitudinal imaging, and the cross-sectional averaged healthy controls (bottom row). (**B**) Box plots show regional tau-PET binding (VTr) at baseline (BL) and follow-up (FUP), with paired t-tests indicating longitudinal increases, consistent with non-corrected results. (**C**) Individual trajectories of GPi tau-PET signal after PVEC over time confirm a robust increase in most patients. Blue markers denote diagnostic certainty levels at baseline and follow-up according to MDS-PSP criteria. Abbreviations: internal part of the globus pallidus (GPi), external part of the globus pallidus (GPe), putamen (PUT), subthalamic nucleus (STN), substantia nigra (SN), dorsal midbrain (DMB), dentate nucleus (DN), dorsolateral prefrontal cortex (DLPFC), and medial prefrontal cortex (MPFC).

**Supplementary Figure 2**


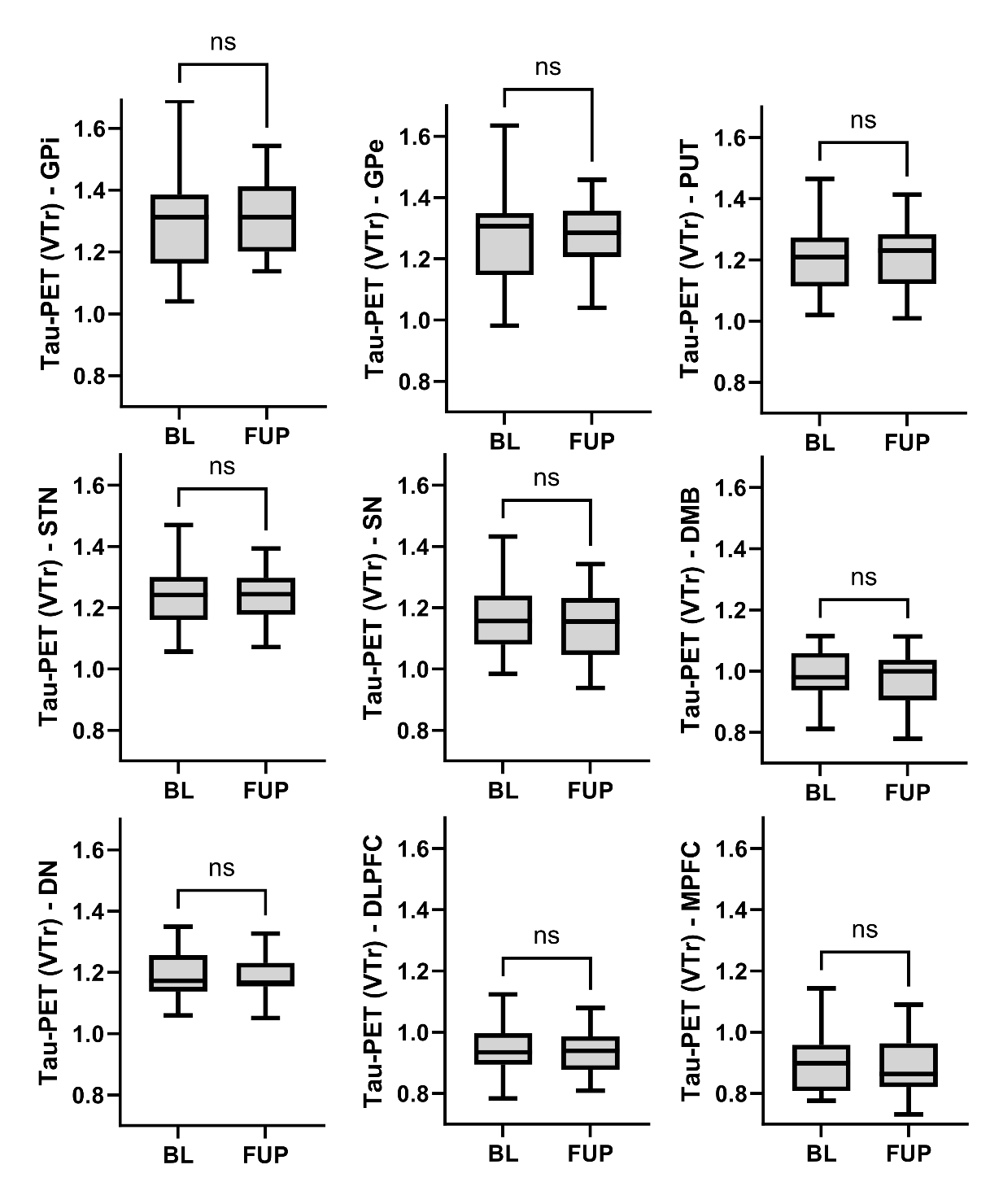
**Supplementary Figure 2**. **Impact of cerebellar reference region on longitudinal tau-PET signal detection.** Box plots show regional tau-PET binding (VTr) at baseline (BL) and follow-up (FUP), using the inferior cerebellum as reference region. Paired t-tests were used to assess temporal differences in VTr. Abbreviations: internal part of the globus pallidus (GPi), external part of the globus pallidus (GPe), putamen (PUT), subthalamic nucleus (STN), substantia nigra (SN), dorsal midbrain (DMB), dentate nucleus (DN), dorsolateral prefrontal cortex (DLPFC), and medial prefrontal cortex (MPFC).

**Supplementary Figure 3**


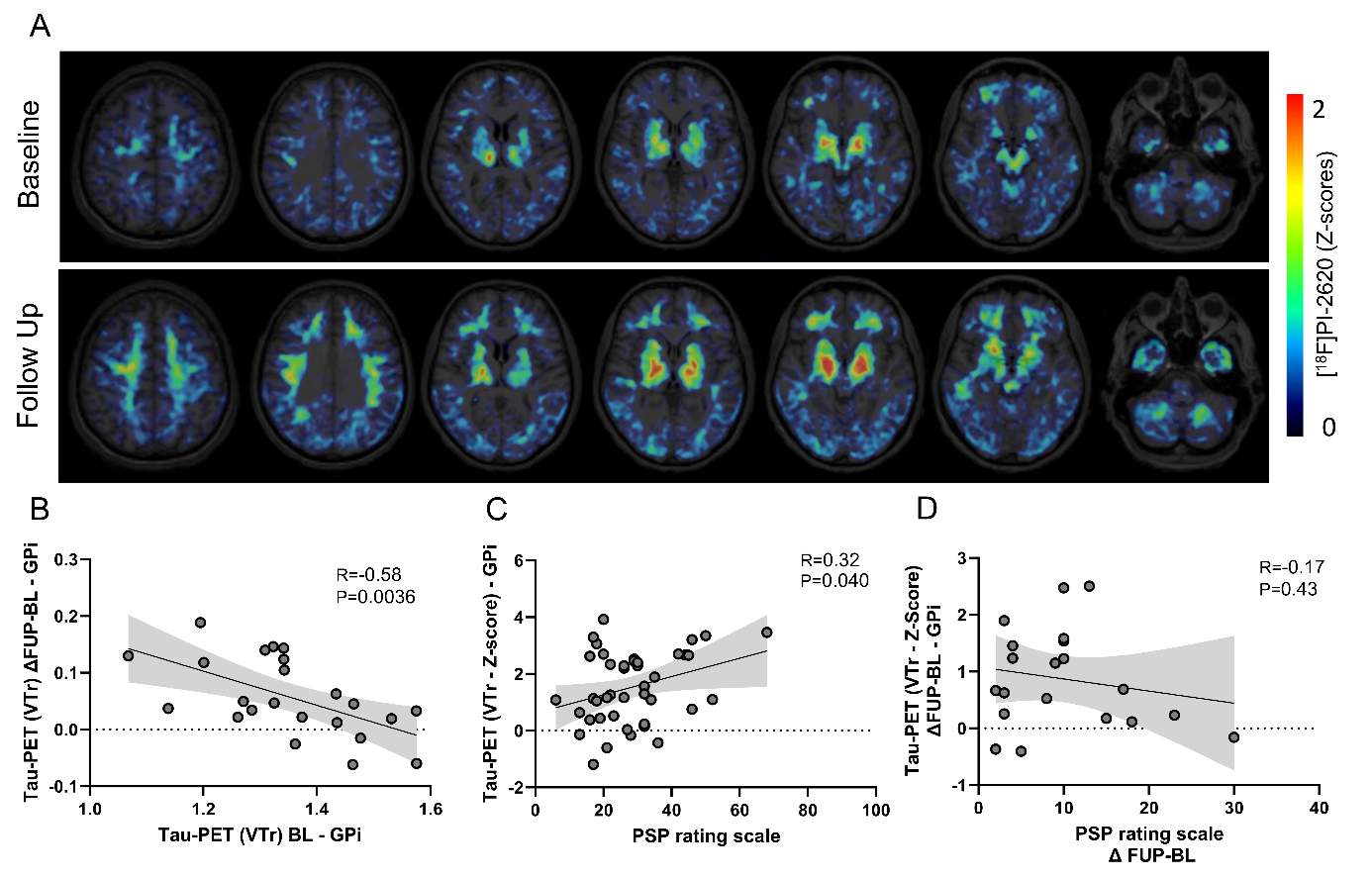
**Supplementary Figure 3** (**A**) Group-averaged axial [^18^F]PI-2620 VTr z-score images at baseline and follow-up after PVEC (**B**) A significant negative correlation between baseline GPi VTr and its longitudinal change (ΔFUP-BL) confirms a ceiling effect in tracer accumulation after PVEC. (**C**) GPi tau-PET z-scores correlate positively with clinical severity, while (**D**) longitudinal changes in tau-PET signal show no significant association with clinical progression. Each dot represents an individual subject (n = 23).

**Supplementary Figure 4**


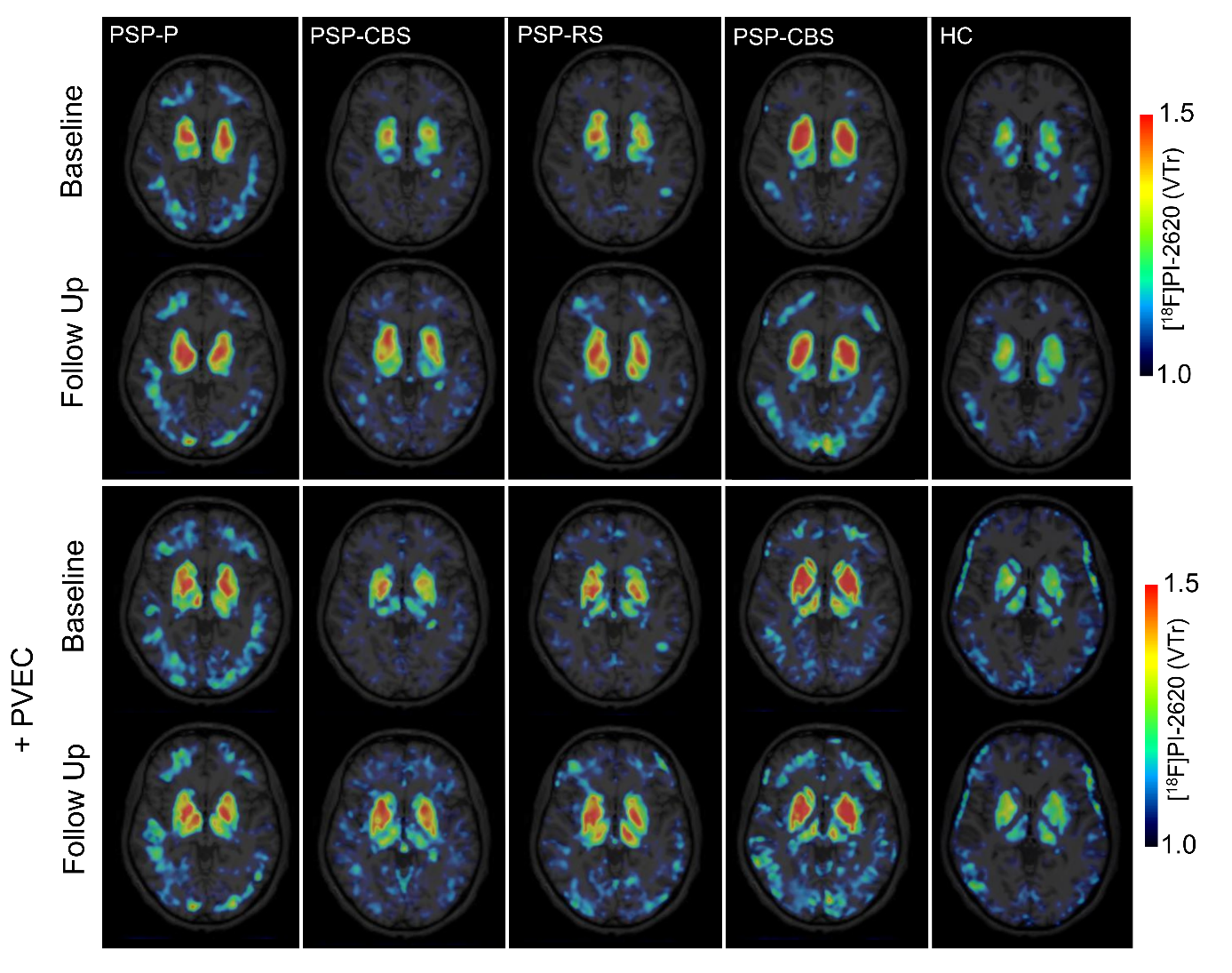
**Supplementary Figure 4.** **Individual [^18^F]PI-2620 tau-PET images before and after PVEC.** Representative axial [^18^F]PI-2620 tau-PET images at baseline and follow-up from individuals with PSP-parkinsonism (PSP-P), PSP-CBS, and PSP-RS, compared to a healthy control (HC), illustrating the consistency of subcortical tracer patterns after application of PVEC.

**Supplementary Figure 5**


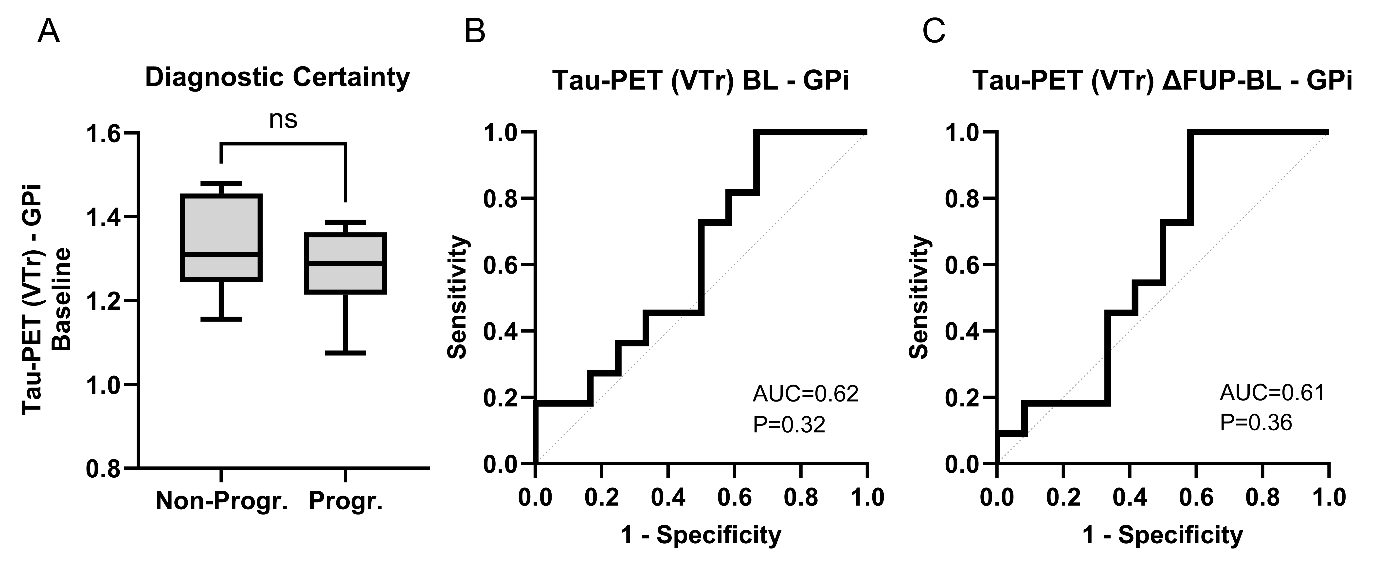


**Supplementary Figure 5. (A)** Box plot depicts baseline tau-PET (VTr) values of the internal part of the globus pallidus (GPi) in patients who progressed to a higher MDS-PSP diagnostic certainty stage (“Progr.”) versus those who remained stable (“Non-Progr.”). (**B, C**) Receiver operating characteristic (ROC) analyses evaluating whether baseline GPi tau-PET (**B**) or longitudinal tau-PET change (ΔVTr) (**C**) discriminated diagnostic certainty progression.
